# Supplementary figures and images for: Extent and context dependence of pleiotropy revealed by high-throughput single-cell phenotyping
Source: PLoS Biol. 2020 Aug 17;18(8):e3000836. doi: 10.1371/journal.pbio.3000836 (PMC7451985; doi:10.1371/journal.pbio.3000836)

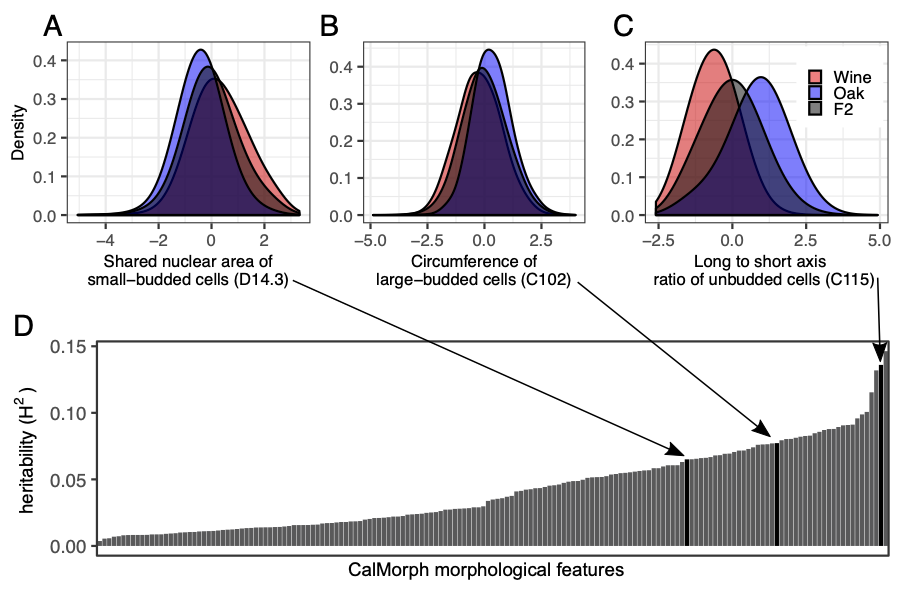

Supplement: S1 Fig — (A–C) Each density plot displays the distribution of phenotype values from yeast cells corresponding to the wine parent (red), the oak parent (blue), or all of the 374 progeny (gray) for the trait listed on the horizontal axis. Trait names in parentheses correspond to those listed in the CalMorph manual [53]. Each distribution represents at minimum 5,000 cells from three replicate experiments; distributions corresponding to progeny strains represent many more cells (70,000–200,000 depending on whether the trait was measured in unbudded, small-budded, or large-budded cells). (D) The broad-sense heritability for each of the 155 morphological features for which QTL were detected. Heritability is low because cell morphology varies across the cell cycle, and so the amount of nongenetic phenotypic variation is high. Data underlying this figure can be found at https://osf.io/b7ny5/. QTL, quantitative trait loci. (TIFF) [file pbio.3000836.s001.tiff]

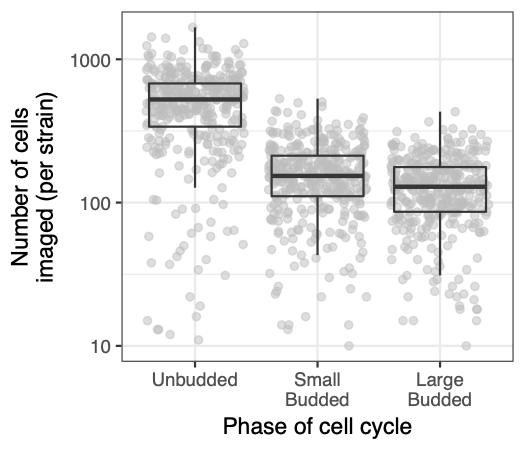

Supplement: S2 Fig — Each point represents, for one of the 374 progeny strains, the number of unbudded, small-budded, or large-budded cells for which images passed filtering. Each box plot shows the median (center line), IQR (upper and lower hinges), and highest value within 1.5 × IQR (whiskers). Data underlying this figure can be found at https://osf.io/b7ny5/. IQR, interquartile range. (TIFF) [file pbio.3000836.s002.tiff]

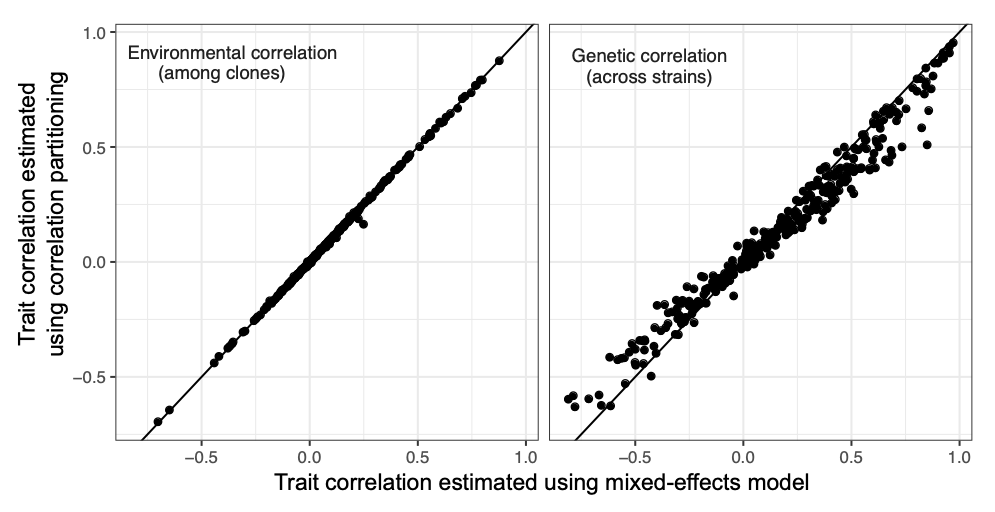

Supplement: S3 Fig — Each point represents one of 350 randomly sampled trait pairs of the 5,645 total. Vertical axes display trait correlations estimated using the correlation-partitioning approach; horizontal axes display trait correlations estimated using a mixed-effect linear model that specifies the variance-covariance structure of the experimental design. Data underlying this figure can be found at https://osf.io/b7ny5/. (TIFF) [file pbio.3000836.s003.tiff]

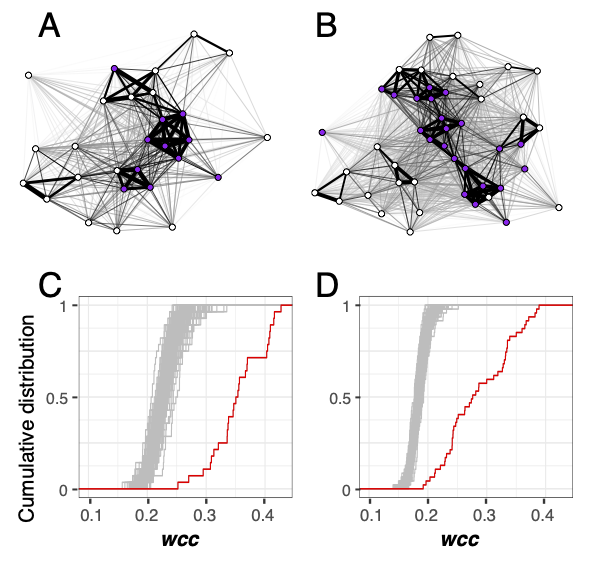

Supplement: S4 Fig — (A–B) Force-directed networks visualizing how pairs of morphological features correlate across clones in unbudded (A) and small-budded (B) cells. Each node represents a single-cell morphological trait. The thickness of the line connecting each pair of nodes is proportional to rW. Node position in the network is determined using the Fruchterman-Reingold algorithm. Purple nodes correspond to traits influenced by a QTL on chromosome 13 containing the HOF1 gene. (C–D) Cumulative distributions of weighted clustering coefficients (wcc) in a network created using measured values of rW (red line) or in 100 permuted networks (gray lines) for traits corresponding to unbudded (C) or small-budded (D) cells. Permutations were performed by sampling rW, without replacement, and reassigning each value to a random pair of traits. Data underlying this figure can be found at https://osf.io/b7ny5/. QTL, quantitative trait loci. (TIFF) [file pbio.3000836.s004.tiff]

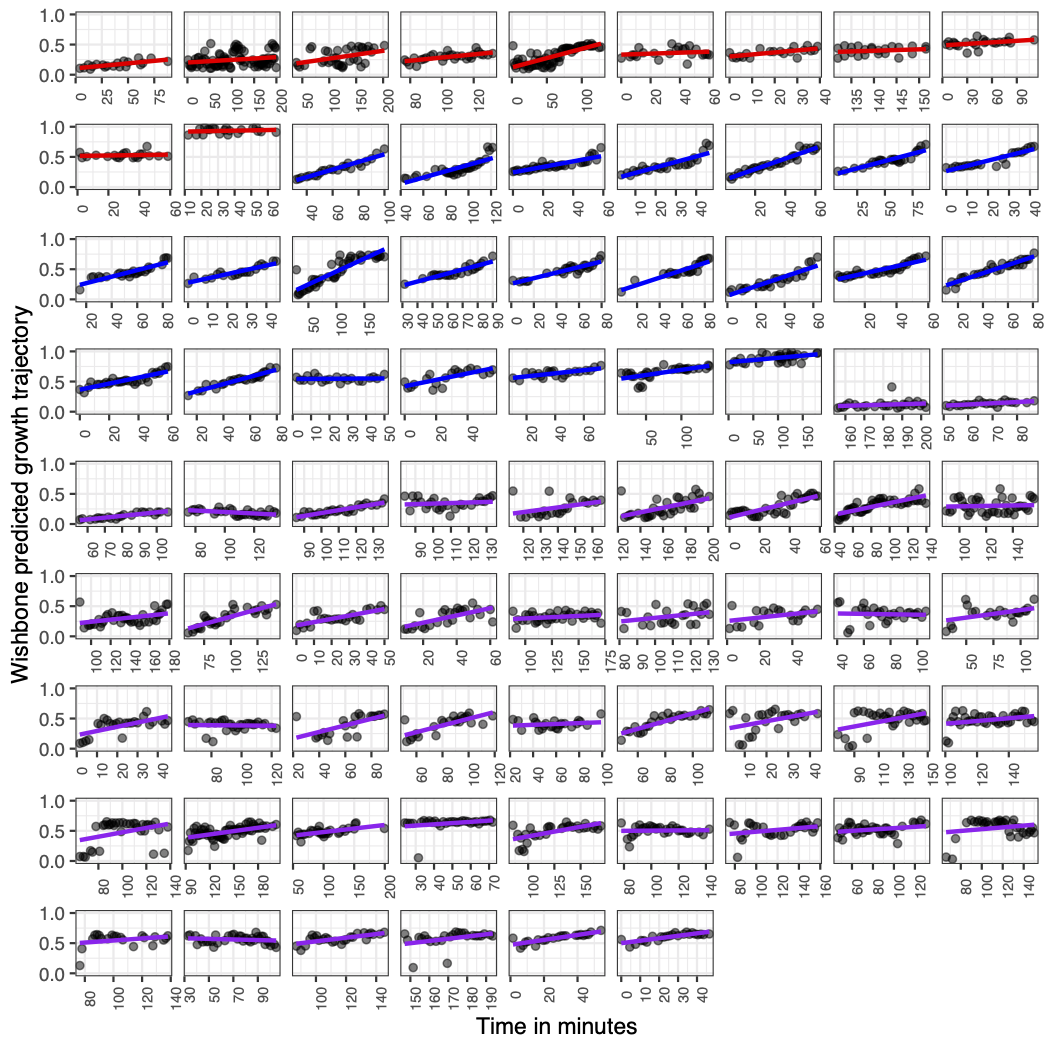

Supplement: S5 Fig — Each point represents a cell image. Horizontal axes display the minute that image was captured during a 3-hour window of exponential growth. Vertical axes display Wishbone’s prediction of how far that cell image has passed through the cell cycle. Linear regression lines are calculated with the “lm” method in the R package ggplot2 [99] and are colored red for images corresponding to unbudded cells, blue for small-budded cells, and purple for large-budded cells. Plots are organized by cell type and then from earliest to latest average predicted progress through cell division. Data underlying this figure can be found at https://osf.io/b7ny5/. (TIFF) [file pbio.3000836.s005.tiff]

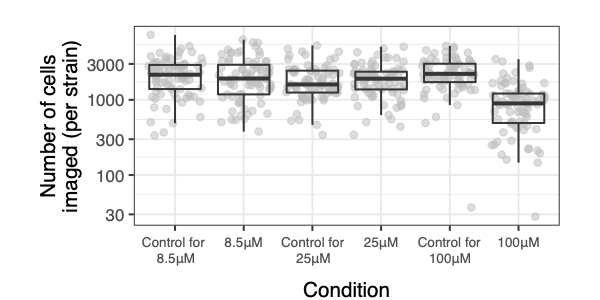

Supplement: S6 Fig — Each point represents, for one of the strains, the number of unbudded, small-budded, or large-budded cells for which images passed filtering. Each box plot shows the median (center line), IQR (upper and lower hinges), and highest value within 1.5 × IQR (whiskers). Data underlying this figure can be found at https://osf.io/b7ny5/. GdA, geldanamycin; IQR, interquartile range. (TIFF) [file pbio.3000836.s006.tiff]
