# Supplementary material for: Extent and context dependence of pleiotropy revealed by high-throughput single-cell phenotyping
Source: PLoS Biol. 2020 Aug 17;18(8):e3000836. doi: 10.1371/journal.pbio.3000836 (PMC7451985; doi:10.1371/journal.pbio.3000836)
Supplement: Attachment — Submitted filename: RTR FINAL.docx [file pbio.3000836.s009.docx]

Below are point-by-point responses to reviewers’ comments. The original reviews are in plain text, and our responses are in **bold**.

REVIEWERS' COMMENTS:


Reviewer #1:

Pleiotropy refers to the common phenomenon that one mutation influences multiple phenotypic traits. In this work, Geiler-Samerotte and colleagues used yeast as a model system to study the biological basis of pleiotropy. They attempted to distinguish between so-called vertical and horizontal pleiotropy and reported detecting both types. They also used mutation accumulation lines to examine whether trait relationships are easily altered by random mutations and found the answer to be yes. They measured 167 yeast cell morphology traits in hundreds of thousands of cells by a high-throughput method, conducted QTL mapping of these traits in 374 segregants from a yeast cross, performed allele replacement experiments for a few genes, and carried out many computational analyses. The amount of work is impressive. However, I have major concerns about the definitions of vertical and horizontal pleiotropy and the method to separate them. In my view, these problems are so fundamental that
their results are hard to interpret if one does not first resolve these conceptual issues.  My detailed comments follow.

**We thank this reviewer for thinking deeply about this topic and for pointing out places we needed to be more clear. We believe we have clarified the definitions of vertical and horizontal pleiotropy, and our method to distinguish them, in the revised manuscript. We have also added experiments to address the reviewer’s major experimental criticism. We reply to the reviewer’s specific concerns below.**

Major comments:

1. Pleiotropy is a characteristic of mutations, not traits. Thus, one must invoke mutations when defining pleiotropy. Yet, vertical pleiotropy was defined and studied in this work without mutations.

**We thank the reviewer for taking the time to explain this misunderstanding. We have made edits to the abstract and to the Results section to clarify our approach. In brief, we agree that one must invoke mutations when studying pleiotropy, and we do so. In our study, first we find specific examples of pleiotropy, i.e. QTLs that each affect more than one trait. Then we try to explain the mechanism underlying these specific examples. It is here that we use variation without mutations to estimate within-strain correlations. The premise is that these within-strain correlations set the baseline expectations that between-strain correlations must exceed to indicate horizontal pleiotropy. We do not take for granted that this premise is sound for the empirical data we gathered, and instead we interrogate the meaning of the within-strain vs. between-strain comparisons in great detail throughout the manuscript (see more on this below).**

Specifically, authors stated that “The key principle is that the distinction between vertical and horizontal pleiotropy lies in whether traits are correlated in the absence of genetic variation. For vertical pleiotropy, the answer is yes: because one trait influences the other or the two share an influence, non-genetic perturbations that alter one phenotype are expected to alter the other.” Here, they are defining and identifying vertical pleiotropy by examining whether two traits are inherently correlated in the absence of mutation. In their words and practice, pleiotropy is now a characteristic of pairs of traits, instead of mutations. They might argue that they are still studying mutations that simultaneously influence two traits. But if one already decides that two traits are
inherently correlated (without even observing a mutation that affect them), what is the relevance of talking about the mutations affecting them? By their definition, such mutations will always be regarded as having vertical pleiotropy.

**Yes, mutations that affect inherently correlated traits will always be regarded as having vertical pleiotropy. For this reason, vertical pleiotropy is often dismissed. But we think it is important to understand vertical pleiotropy, as well as horizontal pleiotropy (the latter having been referred to by others as ‘true’ or ‘genuine’ pleiotropy). We outline the importance of vertical pleiotropy in the introduction with these sentences [lines 141–149]:**

**“It might be tempting to discard vertical pleiotropy as less “genuine” [48] or less important than horizontal pleiotropy, but that would be a mistake because vertical pleiotropy reveals important information about the underlying biological systems that produce the phenotypes in question. Consider the value in identifying yet-unknown factors in heart disease by finding traits that correlate with it, or in understanding where in a system an intervention is prone to produce undesirable side effects. Consider also that the extent and nature of vertical pleiotropy speak directly to the question of modularity: modularity is implied if vertical pleiotropy either is rare or manifests as small groups of correlated traits that are isolated from other such groups.”**

I shall show below that this definition creates inconsistencies. Because horizontal pleiotropy is simply all pleiotropy that is not vertical, the definition of horizontal pleiotropy is also problematic. 

Let us consider the following example. A is the arm length of a human, B is the upper arm length, and C is the lower arm length. So, A=B+C. Clearly, A and B are inherently correlated, because increasing B will increase A. By contrast, B and C need not be inherently correlated.

Now imagine a mutation that simultaneously increases B and C. So, it increases both B and A. Authors will claim that the mutation is vertically pleiotropic on A and B, because A and B are inherently correlated. But in fact, the mutation affects A not entirely through affecting B.

We can imagine another mutation that increases B but decreases C by the same amount such that A is unaffected. Authors would still call this mutation vertically pleiotropic on A and B, because A and B are inherently correlated. But the mutation does not even affect A.

**In this arm example, the reviewer is assuming that B and C vary independently, while A and B do not. Then based on this assumption, the reviewer is able to talk through how the impacts of a mutation would manifest on traits A, B and C. The problem is that we usually do not know which traits vary independently and which are inherently related. This is the problem we address.**

**Our method gives a way of knowing whether B and C are inherently correlated, or the degree to which they can vary independently. Indeed, it might even be that A and B are much more weakly correlated in the arm example than the reviewer suggests. For example, in principle arm morphogenesis could proceed by setting the length of the entire arm (A) followed by subdivision into B and C, in which case B and C would be strongly negatively correlated whereas A and B would be at best weakly correlated unless variation in A is large. That is, in the case of the reviewer’s second hypothesized mutation, which increases B but decreases C, the mutation could do so because B and C are inherently negatively correlated; the within-strain vs. between-strain comparison gives this information. The introduction highlights the point that we need a way to tell which traits can vary independently to better interpret cases of pleiotropy [lines 154–162]:**

**“However, existing methods of distinguishing horizontal and vertical pleiotropy are problematic because judgments must be made about which traits are independent from one another. Such judgments differ between researchers and over time. Indeed, the tomato example can be viewed as a case of horizontal pleiotropy transitioning recently to vertical pleiotropy as knowledge of the underlying system advanced.**

**In this study, we propose and apply an empirical and analytical approach to measuring pleiotropy that relies far less on subjective notions of what constitutes an independent phenotype…”**

This example illustrates the point that pleiotropy has to be defined for mutations rather than pairs of traits without considering specific mutations.

**As explained above, we agree that pleiotropy has to be defined for mutations. Although we study the correlations between all pairs of traits, we only invoke the term ‘pleiotropy’ when describing the action of a QTL. We have scanned the text to look for areas where this distinction could be made more clearly and have edited the text accordingly. Specifically, we edited the following excerpts of the text (relevant changes are in italics):**

**“We focus first on vertical pleiotropy by analyzing correlations that exist in the absence of any genetic differences (*r*_W_). In the analyses that follow, when we refer to *r*_W_ (or *r*_B_), we mean the magnitude of the correlation, as the sign has no relevance for arbitrary pairs of traits. The distribution of *r*_W_ across traits *that are influenced by the same QTL* reflects the degree *to which that QTL acts via* vertical pleiotropy. The overall pattern of *r*_W_ values (*i.e.*, whether there are isolated clusters of highly correlated traits versus a densely interconnected network of traits) reflects the modularity of the underlying biological system.” [lines 410–417]**

**“A test of this hypothesis is to ask whether traits *that are jointly affected by the same QTL* are correlated in the absence of genetic differences.”**

The tomato example in the Introduction can make a similar point. The observation that chloroplasts affect coloration and sugar accumulation does that mean that a mutation that affects coloration and sugar accumulation must affect chloroplasts first.

In other words, it is possible to have some mutations with vertical pleiotropy and some other mutations with horizontal pleiotropy on the two traits of coloration and sugar accumulation.

**Yes, we agree. If multiple genetic variants influence the same pair of traits, some variants could act via vertical pleiotropy and others via horizontal pleiotropy. In other words, a genetic variant acting through horizontal pleiotropy may affect two traits that are already somewhat correlated. This variant would not create a trait correlation where none previously existed, but would instead strengthen the existing correlation, causing *r*_B_ to exceed *r*_W_. We give examples in the text of particular QTL alleles that strengthen trait correlations [lines 627–638, Fig 5B].**

Their definitions of vertical and horizontal pleiotropy and the method for separating them are thus problematic. With this fundamental conceptual flaw, the entire manuscript collapses.

**We appreciate the reviewer taking the time to introduce these examples so that we can think deeply about how to make the core issue our study addresses clearer to our readers. Pleiotropy is a complicated topic and our goal is to provide an approach that makes it more tractable. We have made changes throughout the manuscript for clarity, including large changes to the abstract.**

2. Even under the authors’ definitions of vertical and horizontal pleiotropy, their method of detecting vertical and horizontal pleiotropy is problematic. Specifically, they examine the covariation between two traits among isogenic cells only in the same environment. It is puzzling why they do not examine trait covariation across several environments. This would definitely increase the power of detecting covariation.

**We thank the reviewer for this suggestion. We now include data and a new figure (Fig 7) comparing trait covariation across several environments (a drug gradient), as the reviewer suggests. Our main conclusion is that environments change trait correlations. This result reinforces our conclusion from analyses of cell-cycle state and of different genetic backgrounds that trait correlations are highly context dependent. The result also suggests that correlations across environments are not a good measure of inherent relationships, as we state in the text [lines 724–728]:**

**“We conclude that looking across diverse environments is not a good way to understand the inherent relationships between traits that exist in a single environment. Indeed, previous studies of pleiotropy have treated growth parameters in different environments as different traits (81) rather than as a way to estimate inherent trait correlations.”**

Related to the above point, they did a permutation test in the paragraph starting line 491 in order to see if rW is underestimated. They concluded that it is not, but this is a misinterpretation. The result only means that when there is no difference between groups, rB is not bigger than rW. It does not mean that rW is not underestimated.

**We agree, and indeed we ultimately concluded that *r*_W_ is underestimated. As we state in the text, one reason *r*_W_ could be underestimated is if single-cell measurements are noisier than group-level averages. The permutation test rules out this possibility by suggesting that “measurement noise does not meaningfully obscure *r*_W_” [line 505]. However, we go on to test three additional reasons that *r*_W_ might be underestimated, and find that *r*_W_ is sometimes underestimated due to variation in cell morphology across the cell division cycle.**

3. I suggest that authors reanalyze their data using principal component traits, which are orthogonal to one another so are by definition not correlated. If a mutation affects two or more such traits, it is genuinely (or horizontally) pleiotropic. A critical question is what type of data one should use to define principal component traits. One choice would be morphologies of isogenic cells under the same environment. Another choice, which I think may be more reasonable, would be morphologies of isogenic cells under different environments.

**Using principal component analysis to understand the correlation structure of phenotype data is not uncommon. However, it does not partition the correlation into a genetic and a non-genetic component. Indeed, because trait correlations are hierarchical, as our study overwhelmingly demonstrates, application of principal component analysis is not at all straightforward and applying it to the individual-cell data would not be appropriate.**

**We have made several edits to explain the differences between our method and principal components analysis, including the addition of the following explanation [lines 401–407]:**

**“This approach differs from that of previous studies of pleiotropy that used principal component analysis (PCA) to understand which traits are correlated (17). Performing PCA on the individual-cell data is not the same as controlling for *r*_W_ because PCA would ignore the strain groupings, which can then dominate the analysis (the classic “heterogeneous subgroup” problem in correlation analysis (72)). As a consequence, PCA can miss cases of horizontal pleiotropy and obscure, rather than reveal, inherent trait relationships.”**

**We also note [lines 398–401] that keeping the original (not necessarily independent) traits allows us to examine the modularity of the morphological traits and to identify cases of horizontal pleiotropy.**

4. Their QTL mapping has only 225 markers and 374 segregants. So, the resolution is poor. Given that, one cannot claim to find a pleiotropic QTL simply from the finding that two traits are mapped to the same marker. The marker is unlikely to be causal for either trait; it is highly likely that the causal variants are different for the two traits. Authors are aware of this caveat so performed an allele replacement experiment in an attempt to prove that the QTL is pleiotropic.  But each replaced segment of DNA contains the coding sequence of one gene plus 1 kb flanking sequence, amounting to about 2 kb in total. This sequence would have about 12 SNPs given the divergence of the two strains (0.006). In other words, even when they find that the replaced allele can explain morphological differences between the two strains, there is no guarantee that there is a pleiotropic mutation here (they correctly pointed out in the beginning of Introduction that pleiotropy is a feature of mutations, not genes). Furthermore, the allele replacement was done for only two genes, while 37 QTLs were found “pleiotropic”. While I understand the difficulty to find the causal genes and mutations of a phenotypic variation, the lack of verification of true pleiotropy of QTLs reported here means that all subsequent analyses are compromised, because they all depend on the unproven and most likely wrong assumption that the QTLs are pleiotropic.

**We agree this is an issue with many studies of pleiotropy and QTL studies in general. We added text to the manuscript to discuss this issue. Specifically, we now describe the number of genes within each QTL in the following paragraph [lines 298–307] so the reader has the appropriate context:**

**“When a QTL affects multiple traits, it might not mean that variation in a single gene is contributing to variation in these traits but instead that linked genes are contributing to variation in distinct, individual traits. We partitioned genotype-phenotype associations on the same chromosome into separate QTL when they were greater than 5 cM apart, except in genome regions where many genotype-phenotype associations are present and there is no clear break point. A distance of 5 cM corresponds on average to 8 protein-coding genes in the yeast genome. The largest QTL we detected spans 17 cM, which is roughly half the window size utilized in a previous study of this same QTL mapping family (63). This approach reduces but does not eliminate the possibility that QTL represent the action of linked loci.”**

**Many of our most interesting findings are not dependent on whether there are one or more causal variants underlying each QTL. For example, our quantification of the degree to which the inherent correlations between traits cluster those traits into modules (Fig 2) does not rely on pinpointing causal genetic variants. Nor do our findings that the inherent relationships between traits change throughout the cell cycle (Fig 4), across genetic backgrounds (Fig 6), and across environments (Fig 7).**

**Finally, some of our examples of horizontal pleiotropy cannot simply be explained by multiple genetic variants being linked within one QTL. Specifically, the lower panel of Fig 5B shows a case where two traits are correlated in the absence of allelic variation at a QTL site (blue points). We added text to discuss this important distinction in the section of the results pertaining to Fig 5B [lines 653–663]:**

**“One caveat that remains is whether these examples of pleiotropy represent single genetic changes that influence multiple traits, or the presence of multiple nearby genetic variants within each QTL. Our finding that there are two types of horizontal pleiotropy (Fig 5B), provides some insight. The first type of horizontal pleiotropy (Fig 5B; upper row) may result from the presence of multiple genetic variants segregating together because recombination has not broken them apart. However, the second type of horizontal pleiotropy (Fig 5B; lower row) cannot be explained in the same way because a strong trait correlation exists in the absence of allelic variation at that QTL (blue points). Therefore, although multiple closely linked variants might underlie the difference in the trait correlation between the oak and wine alleles of the QTL, they would act at the level of the correlation rather than of individual traits.”**

Minor comments:

5. Authors should mention vertical and horizontal pleiotropy in the title if this will remain as the focal point of the manuscript, because the current title is too general/vague.

**We want to avoid using terms that we have not defined in the title, so do not use the words vertical or horizontal pleiotropy.**

6. Authors mentioned many times that they studied “thousands of trait pairs”. But these thousands of pairs are not independent from one another. There are probably <100 independent pairs here. So, it is better not to emphasize “thousands of trait pairs”.

**We now discuss the issue of having studied redundant trait pairs directly, explaining which conclusions it may affect [lines 393–401]:**

**“The 167 single-cell morphological features we measured represent 5645 pairs of traits (378, 1081, and 4186 pairs of morphological features pertaining to unbudded, small-budded, and large-budded cells respectively). Because some of these traits are related, these thousands of trait pairs are not independent. This dependence prevents us from reliably counting the absolute number of traits that are influenced by vertical vs. horizontal pleiotropy. Still, partitioning correlations into a non-genetic (*r*_W_) and a genetic (*r*_B_) component for thousands of trait pairs enables us to: 1) analyze a network describing the degree to which morphological traits are interconnected or modular, and 2) detect examples of horizontal pleiotropy if they exist”**

7. The last sentence in the first paragraph of Introduction should be removed, because it is not about pleiotropy. Rather, it is about the number of genes affecting a trait. Having this sentence here would only create confusion.

**These issues are related. With a finite number of causative SNPs and large numbers of gene-trait associations, pleiotropy necessarily follows.**

8. Line 149: “If there is modularity…” Authors imply that, without modularity, there cannot be horizontal pleiotropy. This is not true, as the above examples of arm length and tomato show. For this reason, the sentence on line 208 (“Collectively, the results…”) is also questionable.

**The absence of modularity implies complete interconnectedness. In an extreme world where there is no modularity, and every trait is very correlated with every other trait, all pleiotropy would be vertical. In other words, you would expect every genetic variant to influence every trait because all traits are correlated.**

**Intermediate modularity implies that there are strong correlations between some traits and weaker correlations between other traits. The earlier examples of arm length and tomato both demonstrate modularity. In the arm example, “A and B are inherently correlated” but “B and C need not be inherently correlated”. These differing degrees of correlation allows us to distinguish vertical and horizontal pleiotropy. A QTL that influences A and B (which are inherently correlated) is more likely to act via vertical pleiotropy. A QTL that influences B and C (which “need not be inherently correlated”) could potentially act via horizontal pleiotropy, but only if it increases the correlation between these traits above that observed in the absence of the QTL.**

9. Line 226, it would be good to provide the strain names here.

**We have added the strain names to the methods section.**

10. The paragraph starting line 283. How did the authors predict the causal genes?

**We explain this at the end of the previous paragraph [lines 315–317]:**

**“Candidate genes were selected based on descriptions of the single-cell morphologies of their knockout mutants [66] and the presence of at least one non-synonymous amino acid difference between the wine and oak alleles [62].”**

11. It is unclear whether each cell was measured for multiple traits. I got the impression that each cell was measured for all traits belonging to one of the three groups. Is this understanding right? If each cell is measured for only one trait, how did the authors compute correlations?

**This understanding is correct: each cell is measured for all traits belonging to one of the three groups. We edited the text to clarify this point [lines 385–388]:**

**“Unlike the mapping analysis, which considered phenotypes across all three classes of cell type (unbudded, small-budded and large-budded), this correlation-partitioning analysis can only be applied to pairs of phenotypes that can be measured in the same cell.”**

12. Authors used rW=0.2 as the cutoff to determine whether two traits are inherently correlated. What is the justification of using this cutoff? Cannot they use statistical significance in rW (FDR<0.05) as the cutoff? How would their conclusion change when the cutoff change?

**We have largely removed this cutoff from the main text. We no longer use it to select QTL to focus on in Figs 2 and 5. Now we show data from all QTL regardless of their median *r*_W_.**

13. They mentioned “correlation across clones” a number of time. Is this the same as correlation across cells of the same genotype? Please clarify.

**Yes, these are the same thing. We added text to the abstract to clarify this point, and to clarify the key concepts addressed in the manuscript.**

14. Line 398. “In sum, pairs of traits with stronger correlations across clones (higher rW) are disproportionately represented among those influenced by pleiotropic QTL...” This is circular. How could this statement not be true?

**If all pleiotropy were horizontal, this statement would not be true. The correlations present across clones would not predict those present across genetically diverse strains.**

15. Line 502. “Contrary to this prediction, every morphological trait we surveyed varies MORE within strains than between strains.” Is MORE a typo? How could this be true?

**‘More’ is not a typo. There is a great deal of phenotypic heterogeneity among clonal populations of yeast, which was one of the motivations for us to devise a way to finely divide the cell cycle into 48 stages. This phenotypic diversity is greater than that observed across genetically distinct populations. We added a panel to Fig S1 to show the relative amounts of genetic to total phenotypic variation.**

16. Line 779. It is a pure coincidence that the median numbers of traits affected by a QTL are the same in mouse and yeast traits examined. These are very different traits and different organisms. Previous comparisons show no such equality between different types of traits even for the same species (e.g., Fig. 1 in ref. 11 cited).

**We have moved this text to the Results section and qualified it by explaining that the number of traits influenced by a QTL is dependent on statistical power [lines 245–250].**

Reviewer #2:

Geiler-Samerotte et al. addressed a fundamental and very important question on the nature of pleiotropy. Using yeast high-throughput single-cell imaging and quantitative genetics, the authors were able to decouple 'vertical' pleiotropy, caused by inherent constraints on phenotypes that impose correlations between traits, from 'horizontal' pleiotropy, where the action of a mutation or gene affects several traits that are otherwise not necessarily correlated. The approach is based on computing pairwise trait correlations both between (rb) and within (rw) genotypes.

The amount and quality of the experimental work is impressive, and the rigour and thoroughness of the analysis is even more spectacular. The authors applied fine methods to exclude possible artifacts (eg variation of trait-trait coupling during cell cycle) and they convincingly reach important conclusions: both types of pleiotropy exist, they can be decoupled by observing whether rb exceeds rw, and mutations can change trait-trait correlations. This latter conclusion is also reached by observing mutation-accumulation lines where rare cases of trait-trait correlations appeared in the course of neutral evolution. This is a very remarkable observation.

**We thank the reviewer for these positive words about our study.**

I have two requests of revision:

1) All along my reading, I was preoccupied by the possibility that rb could exceed rw due to reduced phenotypic variation within strains. The authors write that this is not the case (line 503) but they should provide a figure supporting this.

**We have added to Fig S1 to provide these data.**

2) In the QTL mapping, a very large number of traits were tested individually for linkage. Text and methods do not clearly state whether the FDR 5% accounts for this multiplicity (it does account for genome-wide multiplicity but phenome-wide, does it?). The permutation test should be better explained.

**We thank the reviewer for pointing out this omission. We added text to the Results section to explain the permutation test and multiple hypothesis testing considerations [lines 259–272]. We now report analyses based on two sets of QTL: 1) the more lenient set that is significant based on per-trait FDR, and 2) a more stringent set that corrects for multiple hypothesis testing across traits. Qualitative results are the same for the two sets, and we report the effects on the quantitative results at several places in the manuscript, including in Fig 1, to which we added a color scale to distinguish the QTL that are detected after correcting for multiple hypothesis testing.**

Suggestions: line 131, change 'fair' by 'appropriate'; line 260, change 'individuals' by 'cells'.

**We have made these changes.**

Congratulations and thank you for this nice, well-written and helpful contribution to the field of genetics and evolution!


Reviewer #3:

This paper use a yeast-based model to investigate the extent of pleiotropy influenced by related traits (vertical) and by genetic differences (horizontal) . Using a single cell assay they produce an impressive dataset that enable the authors to explore interesting questions related to pleiotropy. 

**We thank the reviewer for these positive words about our study.**

MAJOR COMMENTS
- To further explore the pleiotropy concept, an unrelated phenotype should have been analysed (e.g. growth, drug resistance). The same cohort of segregants has been phenotyped for sporulation and used for QTL mapping. I’m surprised the authors did not look at pleiotropy in this (probably) unrelated phenotype vs. the one they measured and performed a comparison with the QTLs detected there (e.g. different QTLs are expected if no pleiotropy observed).

**We thank the reviewer for this suggestion and added this analysis to the first sub-section of the Results [lines 280–283].**

- The MA Lines (MALs) results are surprising. If I understood correctly, the MALs  are diploids, so if most mutations are heterozygous, how can they affect so much the phenotypes (text lines 737-747).

**Although the mutations were accumulated in diploids, the MA lines have since been sporulated and made haploid. We study the morphology of the resulting haploid strains. We added text to the Methods section to clarify this point [lines 1044–1045].**

These extreme cases should be look more in details, e.g. what mutations are in these MALs? Can we exclude other events that occur at much higher rates (e.g. aneuplodies) can explain this?

**We agree that these are potentially very interesting mutations, and note this in the Results section [lines 701–702]. We added the following sentence to the text in this section as well [lines 694–697]:**

**“Mutations that alter trait correlations are not necessarily the result of rare events such as aneuploidies or copy number variants; all five strains highlighted in Fig 6 do not possess these types of mutations and instead possess at least one single nucleotide mutation [56].”**

MINOR COMMENTS
- The collection of segregants has a gene, sps2 tagged with GFP, does this interfere with the phenotype measured here?

**Sps2 is a reporter of sporulation. Yeast cells in our study are not sporulating, so this gene is not expressed.**

Exact genotype of the strains should be reported to clarify this aspect.

**We have added this information to the Methods section.**

- The collection of MALs should be briefly described (e.g. genotype, genetic background) etc. I guess is derived from lab strains, which is not an ideal comparison with the collection of segregants.

**We added this description to the Methods section.**

- Add grey scale to figure 1a, to indicate effect size, what about chromosome I and III? These should be plotted. Would this figure be more clear as a heat map (markers x traits), with colours reflecting QTL effect size?

**We prefer the original layout of this figure (not a heat map), but have made the other suggested changes.**

- Are there vertically pleiotropic traits that are background dependent? If yes, can QTL be mapped

**Our results suggest that genetic differences can alter trait correlations, and we agree that attempting to map such differences is a promising future direction, but it is beyond the scope of this paper.**

- The sample size is not super big, how this could affect the conclusion of the # of pleiotropic QTLs?

Do you see a correlation with their effect sizes (e.g. stronger QTLs are more pleiotropic?).

**We have added text to the first sub-section of the Results to explain how we are less interested in conclusions regarding the number of pleiotropic QTL and more interested in understanding the mechanisms driving the pleiotropy that we detect.**

**Any QTL-mapping experiment will have a limit of detection such that QTLs with very small effects on traits are invisible. As the reviewer implies, to the extent that we miss some QTLs, we would underestimate the number of pleiotropic QTLs. However, for our QTL analysis, we assayed hundreds of thousands of cells over 374 strains, and we found a large number of QTLs, suggesting our analysis did not suffer from a severe lack of power.**
